# Ascending colon carcinoma presenting as ileocecal intussusception in an adult—a case report with review of literature

**DOI:** 10.1093/jscr/rjaf110

**Published:** 2025-03-05

**Authors:** Areeba Khursheed, Syed A A Rizvi, Wasif M Ali, Mohammad J Hassan, Manzoor Ahmad, Imad Ali

**Affiliations:** Department of General Surgery, JNMCH, AMU, Uttar Pradesh 202002, India; Department of General Surgery, JNMCH, AMU, Uttar Pradesh 202002, India; Department of General Surgery, JNMCH, AMU, Uttar Pradesh 202002, India; Department of General Surgery, JNMCH, AMU, Uttar Pradesh 202002, India; Department of General Surgery, JNMCH, AMU, Uttar Pradesh 202002, India; Department of General Surgery, JNMCH, AMU, Uttar Pradesh 202002, India

**Keywords:** right hemicolectomy, mucinous adenocarcinoma case report

## Abstract

Intussusception is telescoping of a proximal segment of gastrointestinal tract within the lumen of another. Intussusception is a common presentation in pediatric population and present with a classic triad of abdominal pain, bloody diarrhea and a palpable mass. However, intussusception in adults is a rare entity accounting for only 5% of the total cases and is the underlying cause for 1%–5% of all the cases of intestinal obstruction. The adult population caters a varied nonspecific symptoms making diagnosis challenging. We present the case of 32-year-old woman who presented with abdominal pain, altered bowel habits and weight loss. The patient’s clinical presentation, familial history of colonic malignancy underscores the importance of considering malignancy as the underlying cause. A contrast enhanced computed tomography (CECT) whole abdomen was done to diagnose the condition. The patient underwent right hemicolectomy and had an uneventful postoperative period. Histopathological findings were consistent with mucinous adenocarcinoma of the caecum.

## Introduction

First reported in 1674 by Barbette of Amsterdam [1, 2] and further presented in a detailed report in 1789 by John Hunter [3] as “introssusception”, intussusception is defined as telescoping of the proximal segment of the bowel into the other [4]. Although the exact mechanism of intussusception is unclear, it has been hypothesized to result from the altered normal peristalsis in the bowel, most often secondary to the pathological lead point [5]. Ileocolic intussusception in adults is remarkable as 100% cases have malignant lead point, namely cecal adenocarcinoma involving ileocecal valve [5].

The clinical presentation in adults usually encompasses nonspecific symptoms such as abdominal pain, palpable abdominal mass and altered bowel habits with majority of cases in adults are reported to be chronic, constant with partial obstruction. The choice of imaging modality is important in assessment of the underlying cause. Classic finding appreciated on computed tomography (CT) scan include “target sign” due to anatomical configuration of the outer intussuscipiens and the central intussusceptum creating a bowel-within-bowel appearance [6, 7]. Here we present an interesting case of a 32-year-old female with chronic ileocecal intussusception secondary to mucinous adenocarcinoma acting as a pathological lead point.

A 32-year-old Asian woman with no known comorbidities presented to our hospital with 6 months history of vague abdominal pain, weight loss and altered bowel habits. The patient denied any history of nausea, vomiting, or bloody stools. The patient had no history of colonic or any other malignancy in the family. Also, the patient had no prior medical evaluation.

On physical examination, a palpable firm mobile lump in the right iliac fossa was appreciated. Other systemic examinations were unremarkable.

Biochemical investigations were within the normal range expect the evidence of mild nutritional anemia owing to the social status of the patient. The serum carcinoembryonic antigen (CEA) was 1.76 ng/ml.

**Figure 1:**
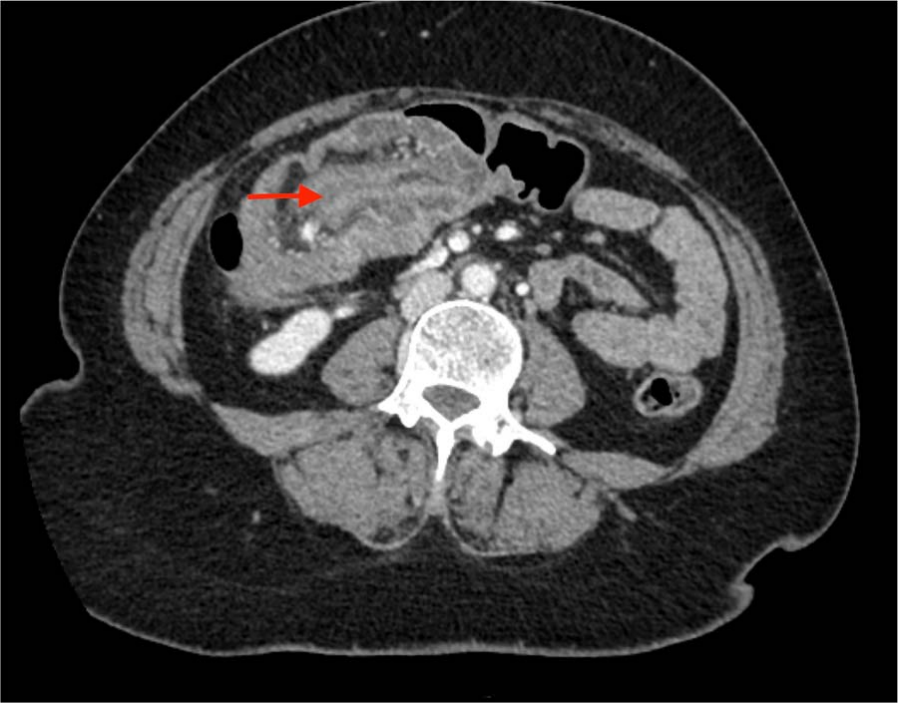
Contrast enhanced CT of the abdomen showing bull’s eye sign (arrow) suggestive of ileocolic intussusception.

**Figure 2:**
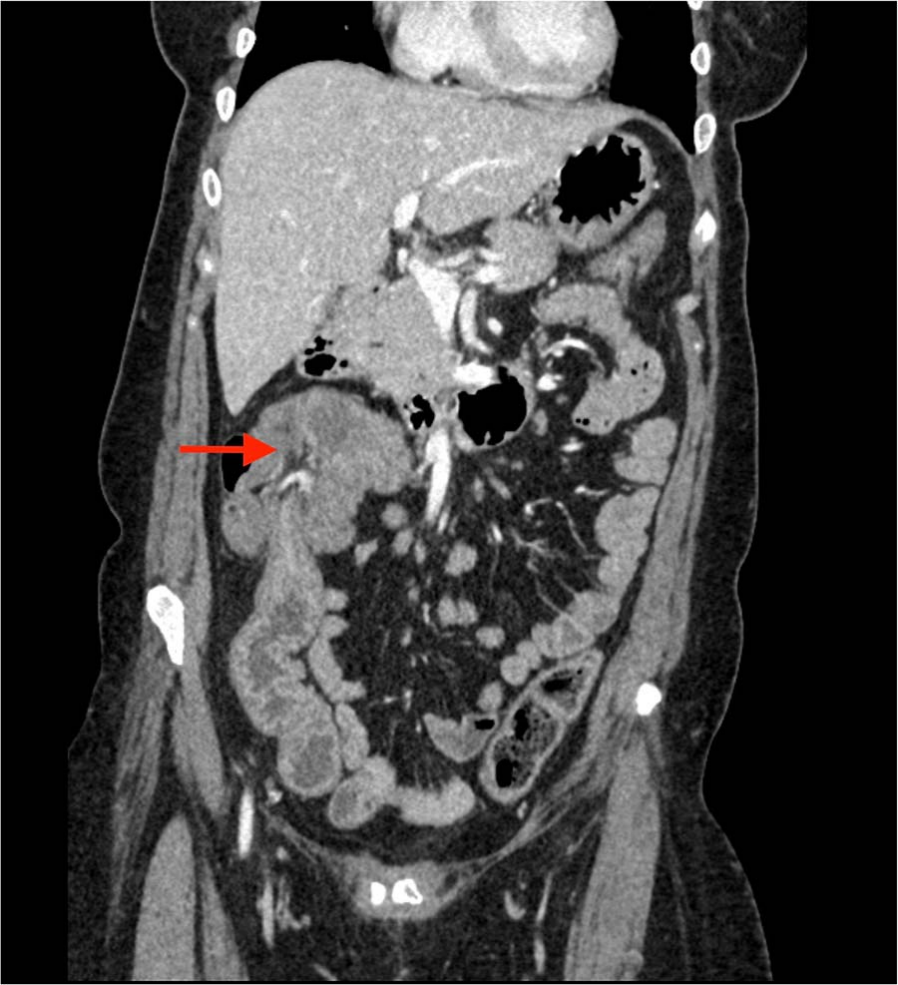
Contrast enhanced CT of the abdomen showing an arrow pointing to the region of ileocolic intussusception.

A CECT scan of the abdomen and pelvis revealed circumferential heterogeneously enhancing mucosal wall thickening involving caecum and ascending colon with pericolic fat stranding and ileocolic intussusception along with multiple enlarged heterogeneously enhancing necrotic lymph nodes in right iliac fossa and pericolic region (Figs 1 and 2).

**Figure 3:**
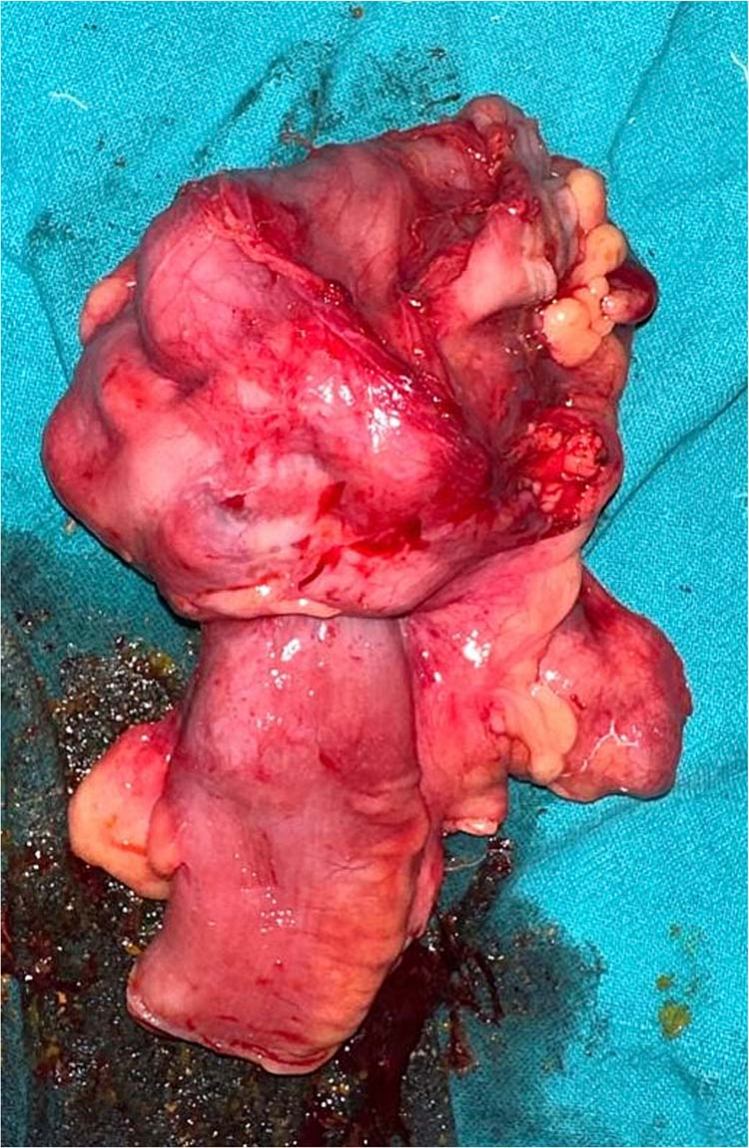
Section of resected specimen showing telescoping of distal ileum into cecum due to a lobulated mass.

The patient was planned for elective surgery and underwent midline laparotomy. Upon exploration, a long 6 cm segment of ileocolic intussusception was identified along the evidence of chronic inflammation. Because of suspicion of malignancy, manual reduction of intussusception was not done. Multiple enlarged mesenteric lymph nodes were seen. The entire abdominal cavity and all the solid organs diligently inspected to rule out any evidence of metastasis. A right hemicolectomy with mechanical end to end ileo-transverse anastomosis was performed and specimen sent for histopathological examination (Fig. 3).

**Figure 4:**
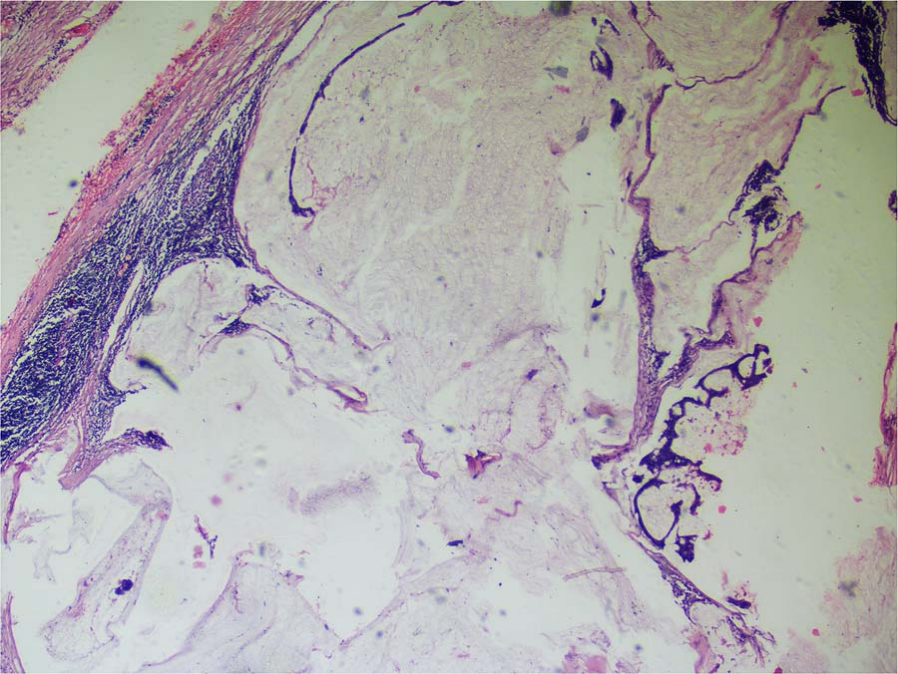
Hemotoxylin and eosin ×100: Section shows atypical cells floating in lates of extra cellular mucin.

**Figure 5:**
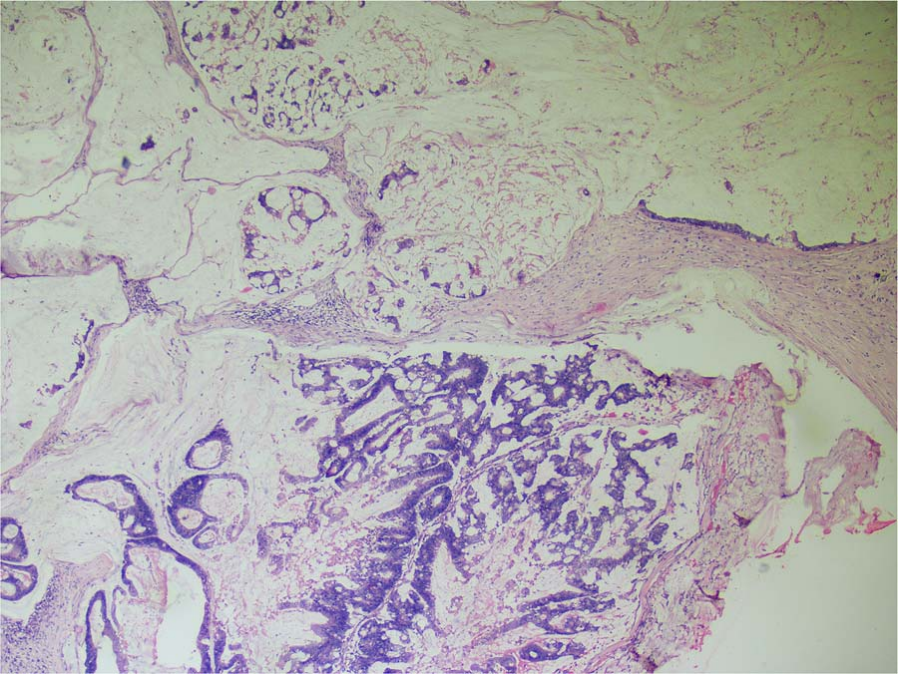
Hematoxylin and eosin ×40: Section shows lymph node effacement of by pool of mucin and few atypical cells and the periphery show compressed lymphoid tissue.

On gross examination, growth seen involving ileocecal junction extending upto caecum measuring 6.5 × 6.0 × 4.3 cm. Cut section showed solid creams white firm to friable areas with mucoid consistency at places. A nodular growth is identified in the attached mesentery measuring 3.5 × 2 × 2 cm. On cut section solid white areas are seen along with focal area showing mucoid consistency. The histopathological report concluded mucinous adenocarcinoma of caecum, pT3N1MX, with evidence of lymphovascular invasion. The tumor invaded through the muscular propria layer (Figs 4 and 5). Additionally, acute appendicitis was found.

Postoperative period was uneventful. Because of the lymph-vascular involvement, the oncology committee decided to initiate adjuvant chemotherapy treatment with CAPEOX regimen.

During the follow-up visits, the patient has fully recovered and is in good health.

## Discussion

Intussusception is described as telescoping of the proximal segment of the bowel into the adjacent segment [4]. Anterograde intussusception occurs when a proximal part of the bowel slides into a distal part, leading to mechanical obstruction and subsequent bowel ischemia [8, 9]. Retrograde intussusception, which is less common, can also occur in certain cases such as post-Roux-en-Y gastric bypass surgery. In this form of intussusception, the distal part of the intestine invaginates into the proximal part.

Intussusceptions have been classified into four categories: (i) entero-enteric, (ii) colo-colic, (iii) ileo-colic, and (iv) ileo-cecal [2].

Intussusceptions have also been classified according to etiology (benign, malignant, or idiopathic). Malignancies are associated with 30% of small bowel intussusception and 66% of large bowel intussusception [2].

In adults, the clinical presentation of intussusception is usually nonspecific chronic with partial obstruction [9]. The most common presenting symptom is abdominal pain with associated symptoms consistent with partial obstruction: nausea, vomiting, obstipation, gastrointestinal bleeding, change in bowel habits, constipation, or bloating. Wang *et al.* found abdominal cramping pain in nearly 80% of patients as a leading symptom [10].

CT is one of the most valuable modality for diagnosis with accuracy of 58%–100% [11].Recently, Ciftci found in a small study of six patients that CT was ideal for the diagnosis of intussusception [12]. Additional valuable information, such as metastases or lymphadenopathy, is readily obtained by CT and may point to an underlying pathology.

Surgical approach, which remains the cornerstone of the treatment can either be traditional or through the minimal invasive approach. Regardless of the approach, the intussusception must be successfully identified and then carefully reduced (in children) or resected (adults). When preoperative colonoscopy, imaging studies, or intraoperative appearance strongly suggests the presence of malignancy, reduction is ill advised and the entire segment should be resected en bloc. In these cases, efforts should be made to resect the unreduced intussusception using oncologic principles so as to minimize risk of spillage and contamination of the abdominal cavity with cancer cells; lymphadenectomy of the major draining vessel(s) should be performed obtaining ≥12 lymph nodes to facilitate proper prognosis and chemotherapeutic treatment recommendations.

## Conclusion

Intussusception in adults is infrequent and usually occurs secondary to the pathological lead point. Multiple diagnostic modalities are often deployed for prompt diagnosis out of which abdominal CT scan is considered most sensitive and feasible noninvasive radiological modality for diagnosis. However histopathological examination is warranted for the confirmation. So a high index of suspicion for underlying malignany in adults and prompt management with proper expertise ensuring adequate surgical resection of malignant pathology is necessary to improve the overall survival of the patient.

## References

[1] De Moulin D, Paul Barbette MD. A seventeenth-century Amsterdam author of best-selling textbooks. Bull Hist Med 1985;59:506–14. 3912022

[2] Marinis A, Yiallourou A, Samanides L, et al. Intussusception of the bowel in adults: a review. World J Gastroenterol 2009;15:407–11. 10.3748/wjg.15.407. 19152443 PMC2653360

[3] Noble I . Master surgeon: John hunter. J Messner: New York 1971;12:185.

[4] Domínguez Páez C, Salazar Andrade JA, Mendoza Tagle DI, et al. Ileocecal intussusception as presentation for ascending colon carcinoma. Case Report Int J Surg Case Rep 2023;108:108439. 10.1016/j.ijscr.2023.108439. 37413757 PMC10382818

[5] Elm'hadi C, Tarchouli M, Khmamouche MR, et al. Intestinal intussusception in a young women: unusual cause and specific management. World J Surg Oncol 2015;13:252. 10.1186/s12957-015-0660-0. 26289057 PMC4545985

[6] Chiang JM, Lin YS. Tumor spectrum of adult intussusception. J Surg Oncol 2008;98:444–7. 10.1002/jso.21117. 18668640

[7] Marinis A, Yiallourou A, Samanides L, et al. Intussusception of the bowel in adults: a review. World J Gastroenterol 2009;15:407–11. 10.3748/wjg.15.407. 19152443 PMC2653360

[8] Zubaidi A, Al-Saif F, Silverman R. Adult intussusception: a retrospective review. Dis Colon Rectum 2006;49:1546–51. 10.1007/s10350-006-0664-5. 16990978

[9] Martin-Lorenzo JG, Torralba-Martinez A, Liron-Ruiz R, et al. Intestinal invagination in adults: preoperative diagnosis and management. Int J Colorectal Dis 2004;19:68–72. 10.1007/s00384-003-0514-z. 12838363

[10] Wang LT, Wu CC, Yu JC, et al. Clinical entity and treatment strategies for adult intussusceptions: 20 years’ experience. Dis Colon Rectum 2007;50:1941–9. 10.1007/s10350-007-9048-8. 17846839

[11] Ciftci F . Diagnosis and treatment of intestinal intussusception in adults: a rare experience for surgeons. Int J Cain Exp Med 2015;8:10001–10005. PMC4538179 26309690

[12] Goh BK, Quah HM, Chow PK, et al. Predictive factors of malignancy in adults with intussusception. World J Sung 2006;30:1300–4. 10.1007/s00268-005-0491-1. 16773257

